# Left Main Stem Compression by Intrapericardial Paraganglioma Associated With Succinate Dehydrogenase Mutation

**DOI:** 10.1016/j.jaccas.2024.102232

**Published:** 2024-02-02

**Authors:** Xue Wang, Mohsin Gondal, Samer Alabed, Catherine Hill, David Barmby

**Affiliations:** a Division of Clinical Medicine, University of Sheffield, Sheffield, United Kingdom; b Department of Cardiology, Sheffield Teaching Hospitals National Health Service Foundation Trust, Sheffield, United Kingdom; c Department of Radiology, Sheffield Teaching Hospitals National Health Service Foundation Trust, Sheffield, United Kingdom

**Keywords:** cardiac magnetic resonance, computed tomography, coronary angiography, coronary artery bypass, genetics, imaging

## Abstract

Paragangliomas are rare extra-adrenal tumors originating from chromaffin cells. We discovered a large intrapericardial mass confirmed to be a primary cardiac paraganglioma encasing the left main stem coronary artery in a 38-year-old woman who presented with dyspnea and subscapular pain. Genetic predisposition related to succinate dehydrogenase A mutation was identified.

## Graphical abstract

**Figure:**
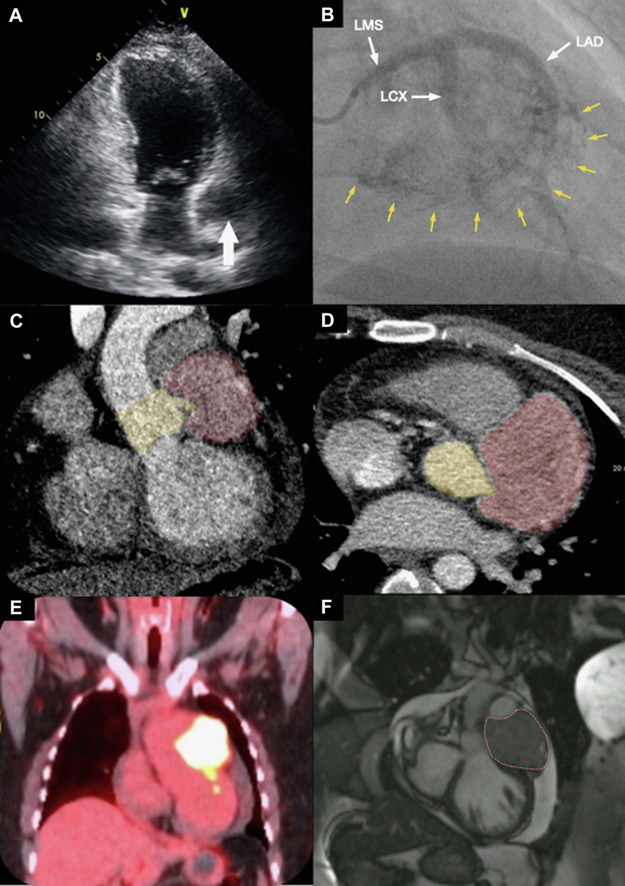

A 38-year-old woman was referred to the cardiology clinic with a 2-month history of mild exertional dyspnea along with sharp left subscapular and axillary pain unrelated to exertion. There was no significant past medical history or family history. Vital signs, physical examination, 12-lead electrocardiogram, and laboratory tests including N-terminal pro–B-type natriuretic peptide were normal. Chest x-ray showed cardiomegaly with an increased cardiothoracic ratio of 0.63. Transthoracic echocardiogram (Figure 1A) demonstrated an area of extrinsic compression of the anterior wall of the left atrium and a 19 mm global pericardial effusion.

**Figure 1:**
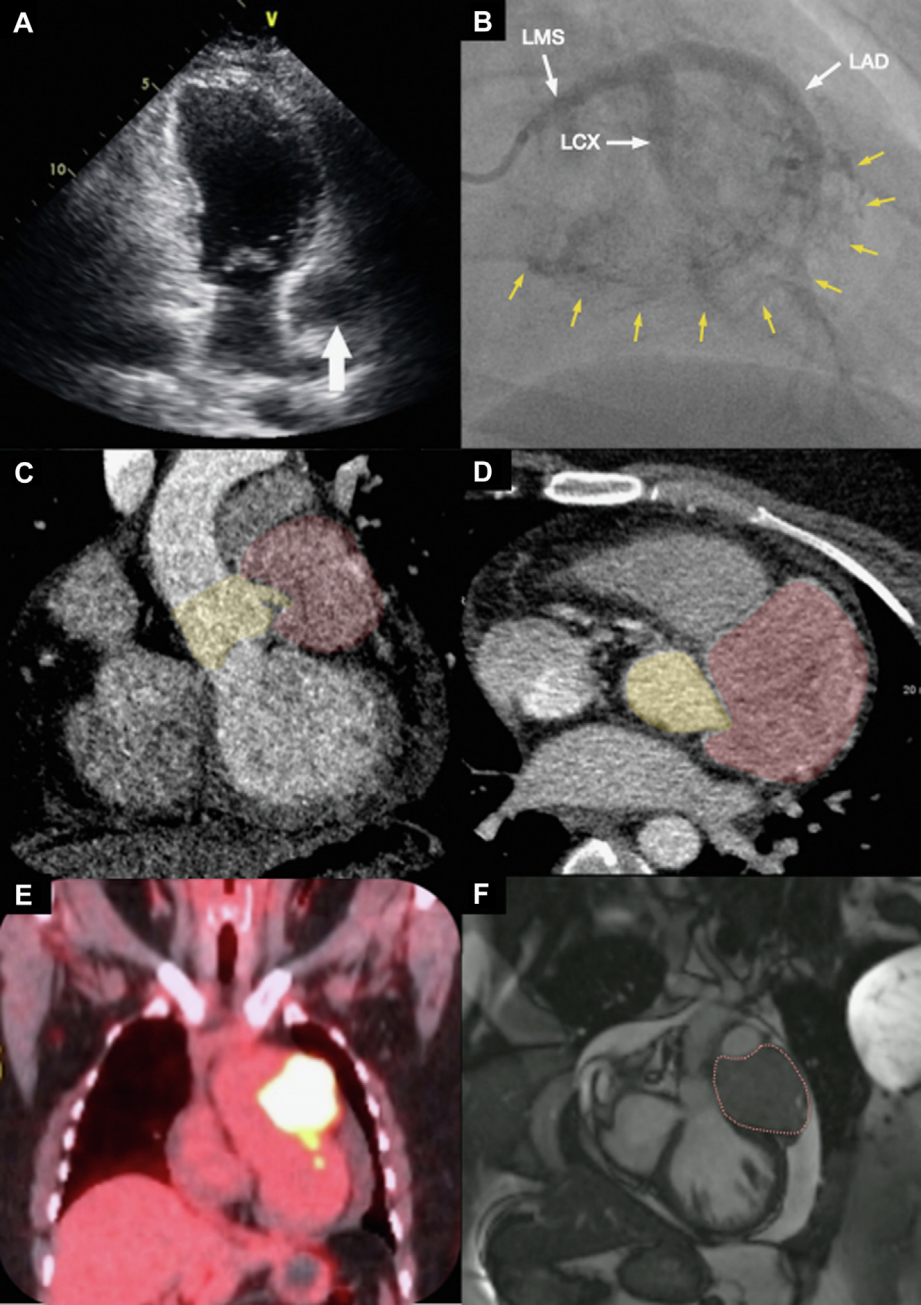
Multimodality Imaging Findings (A) Transthoracic echocardiogram shows an unusual appearance next to the left atrium (arrow) that is suggestive of extrinsic compression. (B) Coronary angiography demonstrating significant contrast blush (yellow arrows) from the highly vascular paraganglioma encasing the left coronary artery and its branches (white arrows). (C and D) Computed tomography thorax coronal and axial images of the coronary arteries showing the mass encasing and compressing the left main stem (LMS) and proximal left anterior descending coronary artery (LAD) (red: mass; yellow: aortic root and LMS). (E) Intense fluorodeoxyglucose F 18 uptake on positron emission tomography–computed tomography. (F) Dashes outline the intrapericardial paraganglioma on cardiac magnetic resonance imaging. LCX = left circumflex coronary artery.

Computed tomography thorax with contrast and gated computed tomography–coronary angiogram revealed a 71 mm × 66 mm × 54 mm heterogeneous vascular soft tissue mass within the pericardial sac, located around the left anterior atrioventricular groove, associated with venous collaterals. The mass was abutting the major vessels (aortic root, pulmonary trunk, and proximal left main branch pulmonary artery) and the surrounding structures (left atrium and the basal anterior segment of the left ventricle). The origin of the left main stem (LMS) coronary artery was encased by the mass (Figures 1C and 1D), due to which the LMS and proximal left anterior descending coronary artery appeared markedly stenosed. Coronary angiography (Figure 1B, Video 1) revealed marked pressure damping on left coronary cannulation secondary to extrinsic compression of the LMS by the mass. Significant contrast blush was noted, indicating a substantial blood supply contribution from the left coronary artery. The mass had intense fluorodeoxyglucose-F18 activity on whole-body positron emission tomography (Figure 1E) with no evidence of distant metastases. It was isointense to myocardium on T_1_-weighted and hyperintense on T_2_-weighted cardiac magnetic resonance imaging, with no definite evidence of myocardial infiltration (Figure 1F, Video 2). The small pericardial effusion was unchanged. An exercise test was performed with a modified Bruce protocol during which the patient developed chest tightening with widespread 2-mm ST-segment depression in the anterolateral leads on the electrocardiogram.

The patient underwent successful resection of the intrapericardial mass with a saphenous vein Y-graft to both left anterior descending coronary artery and obtuse marginal arteries. Histopathology results confirmed the diagnosis of paraganglioma. Plasma metanephrines were normal. Genetic testing confirmed the presence of succinate dehydrogenase (SDH) A mutation.

At 18 months postsurgery, redo coronary artery bypass graft was performed due to graft failure. Seven years since her diagnosis, the patient has remained symptom free with no tumor recurrence.

## Discussion

Cardiac paragangliomas, originating from chromaffin cells of sympathetic ganglia, account for under 1% of all cardiac tumors.^1^ Although rare, the incidence of paragangliomas has increased over time possibly from increased detection with imaging.^2^ This case highlights the importance of multimodality imaging and multidisciplinary involvement in the management of cardiac paragangliomas. Germline mutations in the genes encoding SDH subunits A-D contribute to approximately 20% of paragangliomas/pheochromocytomas, whereas somatic mutations are rarer. Penetrance of SDH mutations is incomplete, with a reported penetrance of 13% for SDH A mutations.^3^ Genetic counselling, especially prior to pregnancy planning, and family screening are recommended.^2^^,^^3^ Complete surgical resection remains the only definitive treatment, and surveillance for recurrence is advised.^2^

## Funding Support and Author Disclosures

The University of Sheffield Institutional Open Access Fund covered the article processing charge. The authors have reported that they have no relationships relevant to the contents of this paper to disclose.

## Appendix

Video 1 Coronary Angiography Coronary angiography demonstrating significant contrast blush from the highly vascular paraganglioma encasing the left coronary artery and its branches.

Video 2 Cardiac Magnetic Resonance Cardiac magnetic resonance coronal view showing the cardiac mass and a small pericardial effusion.
